# New Insights into rice pyrimidine catabolic enzymes

**DOI:** 10.3389/fpls.2023.1079778

**Published:** 2023-02-01

**Authors:** Andrea J. Lopez, Heidy Y. Narvaez-Ortiz, Maria A. Rincon-Benavides, Dania Camila Pulido, Luis Eduardo Fuentes Suarez, Barbara H. Zimmermann

**Affiliations:** Departamento de Ciencias Biológicas, Universidad de los Andes, Bogotá, Colombia

**Keywords:** pyrimidine catabolism, dihydropyrimidine dehydrogenase, dihydropyrimidinase, ßureidopropionase, dihydroorotate dehydrogenase, plants, *Oryza sativa*, abiotic stress

## Abstract

**Introduction:**

Rice is a primary global food source, and its production is affected by abiotic stress, caused by climate change and other factors. Recently, the pyrimidine reductive catabolic pathway, catalyzed by dihydropyrimidine dehydrogenase (DHPD), dihydropyrimidinase (DHP) and β-ureidopropionase (β-UP), has emerged as a potential participant in the abiotic stress response of rice.

**Methods:**

The rice enzymes were produced as recombinant proteins, and two were kinetically characterized. Rice dihydroorotate dehydrogenase (DHODH), an enzyme of pyrimidine biosynthesis often confused with DHPD, was also characterized. Salt-sensitive and salt-resistant rice seedlings were subjected to salt stress (24 h) and metabolites in leaves were determined by mass spectrometry.

**Results:**

The OsDHPD sequence was homologous to the C-terminal half of mammalian DHPD, conserving FMN and uracil binding sites, but lacked sites for Fe/S clusters, FAD, and NADPH. OsDHPD, truncated to eliminate the chloroplast targeting peptide, was soluble, but inactive. Database searches for polypeptides homologous to the N-terminal half of mammalian DHPD, that could act as co-reductants, were unsuccessful. OsDHODH exhibited kinetic parameters similar to those of other plant DHODHs. OsDHP, truncated to remove a signal sequence, exhibited a kcat/Km = 3.6 x 103 s-1M-1. Osb-UP exhibited a kcat/Km = 1.8 x 104 s-1M-1. Short-term salt exposure caused insignificant differences in the levels of the ureide intermediates dihydrouracil and ureidopropionate in leaves of salt-sensitive and salt-resistant plants. Allantoin, a ureide metabolite of purine catabolism, was found to be significantly higher in the resistant cultivar compared to one of the sensitive cultivars.

**Discussion:**

OsDHP, the first plant enzyme to be characterized, showed low kinetic efficiency, but its activity may have been affected by truncation. Osb-UP exhibited kinetic parameters in the range of enzymes of secondary metabolism. Levels of two pathway metabolites were similar in sensitive and resistant cultivars and appeared to be unaffected by short-term salt exposure.”

## Introduction

Pyrimidines are essential biomolecules in all cells. They are required for the synthesis of nucleic acids, and they play additional key roles, participating in the synthesis of lipids and sugars (Garavito et al., 2015; Witte and Herde, 2020). The metabolism of pyrimidines includes (1) the *de novo* pathway for synthesis of pyrimidine nucleotides, in which small precursor molecules are assembled into UMP, (2) pathways for interconversions, and (3) the “salvage” pathways, where pyrimidine bases or nucleosides are recycled by addition of ribose or phosphates (Garavito et al., 2015; Witte and Herde, 2020). Plants and other higher eukaryotes also contain (4) a reductive catabolic pathway to degrade the bases uracil or thymine [Zrenner et al., 2006; Witte and Herde, 2020).

The reductive catabolic pathway consists of three steps, first, the reduction of the bases to dihydrouracil or dihydrothymine, catalyzed by dihydropyrimidine dehydrogenase (DHPD) (EC 1.3.1.1 using NADH, or 1.3.1.2 using NADPH), second, the cleavage of the ring structures to form ß-ureidopropionate or ureidoisobutyrate by dihydropyrimidinase (DHP) (EC 3.5.2.2), and finally the release of CO_2_ and NH_3_ to produce ß-alanine or ß-aminoisobutyrate by β-ureidopropionase (β-UP) (EC 3.5.1.6). In a seminal work by Zrenner et al. (2009), the enzymes from *Arabidopsis thaliana* were localized to the chloroplast (DHPD, *PYD1*), the secretory system (DHP, *PYD2*), and the cytosol (ß-UP, *PYD3*). No phenotypes are observed for knockouts of the corresponding genes, *PYD1*, *PYD2*, and *PYD3*, respectively, under normal growth conditions, suggesting that the pathway pertains to secondary metabolism (Zrenner et al., 2009). However, under nitrogen limitation, increased *PYD* expression, and a significant increase in [^14^C]CO_2_ release from [^14^C]uracil, are observed (Zrenner et al., 2009). These authors suggest that the functions of the pathway are to maintain cellular pyrimidine levels, and to recycle nitrogen for general nitrogen metabolism (Zrenner et al., 2009). Cornelius et al. (2011) show high levels of *A. thaliana* PYD1 transcripts in seeds and during senescence, and note increased transcription levels in the presence of the phytohormone abscisic acid (ABA). Knockouts of the enzyme lead to accumulation of uracil, and overexpression increases growth and produces higher seed numbers compared to wild-type plants.

Understanding the contribution of the individual enzymes to pyrimidine degradation has been hampered by the difficulty of measuring their activities *in vivo*. To address this problem, we produced recombinant proteins corresponding to the three enzymes of the pathway from *Oryza sativa*, and kinetically characterized the second and third enzymes. The recombinant OsDHPD was soluble, but inactive, and is likely to require the presence of an additional chloroplast protein or proteins, as has been suggested previously (Zrenner et al., 2009; Cornelius et al., 2011). The first enzyme is upregulated in the presence of ABA in rice and in Arabidsopsis (Liu et al., 2009; Cornelius et al., 2011). The report that transgenic rice plants with knockdowns of the enzyme have increased salt sensitivity, while plants overexpressing this enzyme have increased salt tolerance (Liu et al., 2009) led us to explore the effects of salinity on the metabolite levels of the pathway.

## Materials and methods

### Reagents and materials

Reagents were from Sigma-Aldrich Products or Santa Cruz Biotechnology. Restriction enzymes and enzymes used for cloning were from New England Biolabs. Azucena rice seeds were provided by the Center for Tropical Agriculture (CIAT), Cali, Colombia. Koshihikari seeds were from the Kitazawa Seed Company, Oakland, USA.

### Sequence analysis

Signal peptide sequences were predicted with TargetP 1.1 (plant network) (Nielsen et al., 1997; Emanuelsson et al., 2000), and PrediSi (http://www.predisi.de/home.html) Multiple sequence alignments shown in the Supplementary material were performed with Clustal Omega (Madeira et al. 2022).

### Expression constructs

RNA was extracted from the leaves of rice seedlings ground in liquid nitrogen using the commercial SpectrumTM Plant Total RNA kit (Sigma). The RevertAid First Strand cDNA Synthesis Kit (Thermo Scientific™) was used to produce cDNA that was quantified using a Thermo Scientific Multiskan GO UV/visible spectrophotometer.

The GenBank sequences DQ102485 (OsDHPD), AK072454.1 (OsDHP), AK060443.1 (Osß-UP), DQ269457.1 (OsDHODH) were used to design PCR primers to amplify the coding sequences for each gene (**Supplementary Table 1**). Adenylated PCR products were ligated into pGEM^®^-T-Easy (Promega, United States) and transformed into *Escherichia coli* DH5-α. Inserts were sequenced at the Universidad de los Andes sequencing facility.

Full-length OsDHPD was subcloned into the BamH1 site of pET19b (Novagen). The full-length recombinant protein was insoluble, so a truncated version, OsDHPD-T, lacking 39 residues on the N-terminus (starting with LSVR), eliminating the chloroplast targeting sequence was subcloned into the BamH1 site of pET19b (**Supplementary Figure 1**). The his-tagged OsDHPD-T had a molecular mass of 44.5 kDa.

We were unable to amplify a full-length sequence corresponding to OsDHP from the cDNA, however we succeeded in amplifying two overlapping fragments, a 741 bp 5’-end fragment, and a 1,179 3’-end fragment, which shared a 300 bp overlap containing an endogenous NcoI site. Each of the fragments was cloned into pGEM^®^-T-Easy. The pGEM^®^-T-Easy -5’-end fragment was digested with NotI (vector site) and NcoI (OsDHP sequence site) and the pGEM^®^-T-Easy -3’-end fragment was digested with NcoI (OsDHP sequence site) and EcoRI (vector site). The restricted DHP fragments were gel-purified and ligated into the modified TOPO™ TA vector, previously restricted with NotI and EcoRI and gel-purfied. The resulting full length DHP-TOPO construct was digested with XhoI and subcloned into the XhoI site of pET19b. The full-length recombinant was insoluble. Two N-terminal truncations were produced to remove signaling sequences, OsDHP-T1 starting with residue 30 (EFCA), and OsDHP-T2 starting with residue 44 (GGDG) (**Supplementary Figure 2**). Both truncated recombinant proteins were soluble, but OsDHP-T2 was inactive. Kinetic studies were performed with OsDHP-T1. The his-tagged recombinant OsDHP-T1 had a molecular mass of 58.2 kDa.

The full-length Osß-UP sequence was subcloned into the BamHI site of pET15b (Novagen), was soluble, and the his-tagged recombinant protein had a molecular mass of 48.9 kDa.

A truncated version of OsDHODH, starting at residue 82 (DEAK), was subcloned into the BamHI site of pET15b. DHODHs from most organisms are expressed as truncated recombinant proteins containing all sequences involved in catalysis, while eliminating N-terminal targeting and membrane associated sequences.

### Expression and purification

N-terminally his-tagged, soluble, recombinant proteins were expressed and purified using the following protocol. Briefly, *E. coli* BL21-CodonPlus(DE3)-RP originating from a single colony that had been transformed with the selected expression construct were grown overnight in Luria-Bretani medium (LB) containing 100 μg/mL ampicillin at 37°C with 200 rpm agitation. A large-scale culture of LB with 100 μg/mL ampicillin was inoculated at 5% with the overnight culture, and grown at 37°C. When the large-scale culture reached an OD_600nm_ of 0.5–0.6, expression was induced with 1 mM isopropyl ß-D-thiogalactopyranoside, and growth was continued for 24 h at room temperature. Cells were then centrifuged at 3,500 x *g* for 15 min at 4°C, and the pellets were stored at -80°C. The medium for expression of OsDHPD recombinant proteins was supplemented with 1 mM uracil and 10 µM flavin mononucleotide (FMN).

The pellet from 0.5 L of bacterial culture was resuspended in purification buffer. The purification buffers were different for the three recombinant proteins, OsDHPD-T (50 mM sodium phosphate pH 7.5, 300 mM NaCl, 5 mM ß-mercaptoethanol, 5 mM imidazole), OsDHP-T1 (50 mM sodium phosphate pH 8, 300 mM NaCl, 5 mM ß-mercaptoethanol, 5 mM imidazole), Osß-UP (100 mM sodium phosphate pH 7.3, 1 mM MgCl_2_, 300 mM NaCl, 5 mM dithiothreitol, 5 mM imidazole). All three buffers also contained the protease inhibitors 1 mM phenylmethanesulfonyl fluoride and 1 mM benzamidine. Cells were lysed by incubating with lysozyme (1 mg/mL) on ice for 2 h, followed by sonication on ice (30 cycles of 20 s each, output control 8, 100% duty cycle) using a 250 Analog Sonifier (Branson). The sonicated cell extract was centrifuged (8,500 x *g*, 1 h, 4°C), and the supernatant was loaded onto a Co^2+^ affinity column (Thermo Scientific) previously equilibrated with purification buffer, washed with purification buffer containing 30 mM imidazole, and eluted with purification buffer containing 250 mM imidazole. Removal of imidazole and buffer exchange was achieved using PD-10 columns (Sephadex G-25-M, GE Healthcare) with buffer (50 mM Tris-HCl pH 8, 150 mM NaCl, and 10% glycerol). The yield of purified protein was as follows, 7.3 mg OsDHPD-T/L culture, 6.1 mg OsDHP-T1/L culture, and 8.7 mg Osβ-UP/L culture.

### SDS-PAGE

Protein samples were fractionated by SDS-PAGE on 12% running gels, with 5% stacking gels. Electrophoresis was performed in a BioRad Mini-Protean II electrophoresis cell for 1 h, at 200 volts, constant voltage. Gels were visualized by staining with Coomassie Blue G-250 dye. **Supplementary Figure 3** shows SDS-PAGE of purified recombinant OsDHPD-T, OsDHP-T2, and Osß-UP proteins.

### Enzymatic assays and kinetic analysis

Purified OsDHODH activity was measured as described by Garavito et al. (2019).

Three different assays were used to test the activity of the OsDHPD-T recombinant protein. In the first assay, activity was measured at 37°C, in the biological direction with 1 mM uracil as the substrate, in the presence of 150 µM NADPH or NADH, monitoring the absorbance at 340 nm, according to Yokota et al. (1994). In the second assay, the reverse reaction was measured using 1 mM dihydrouracil and 150 µM NADP^+^ or NAD^+^, monitoring the increase of absorbance at 260 nm, due to the formation of uracil, according to Dolegowska et al. (2012). In a third assay, normally used for measuring type I dihydroorotate dehydrogenase activity, dihydroorotate was used as the substrate, and 1 mM fumarate and 0.1 mM DCIP, following the reduction of DCIP at 600 nm, according to Zameitat et al. (2004). No activity was observed for the enzyme in any of these assays.

The activity of purified OsDHP-T1 recombinant protein was assayed by measuring N-carbamoyl-ß-alanine using a colorimetric procedure (West et al., 1982; Mejias-Torres & Zimmermann, 2002). Reactions were initiated by adding enzyme to concentrations of 90 - 150 nM to a solution containing 0.1 M potassium buffer, pH 8, 1 mg/mL bovine serum albumin and dihydrouracil, 37°C for 10 minutes, and then quenched followed by an incubation for 120 minutes at 70°C for color development. The concentration of ß-ureidopropionate was calculated from the absorbance at 466 nm with a standard curve. All assays were performed in triplicate, using a Beckman Coulter DU 800 UV/visible spectrophotometer.

The activity of purified Osβ-UP was measured colorimetrically for two substrates at 25°C with a modified version of the Berthelot reaction using salicylate hypochlorite (Bower and Holm-Hansen, 1980). In this assay, the NH_3_ produced by 100 – 200 nM enzyme in 5 – 20 minutes was converted to monochloroamine by a solution of 0.1% sodium hypochlorite in 1.5 M NaOH. Addition of a solution containing 425 mM sodium salicylate, 190 mM trisodium citrate dihydrate, 177 mM sodium potassium tartrate tetrahydrate, 0.84 mM sodium nitroprusside converted the monochloroamine to 5-aminosalicylate, which became oxidized to a blue-green dye absorbing at 650 nm. The NH_3_ concentration was calculated using a standard curve of NH_4_Cl. Absorbance was measured in microplates with a Thermo Scientific Multiskan GO UV/visible spectrophotometer.

Kinetic data for OsDHP-T1, OsßUP, and OsDHODH were fit to the Michaelis–Menten equation v = V_max_∗[S]/(K_m_ + [S]) using GraphPad Prism v7 software.

Protein concentration was measured using the Bradford assay (Pierce) with bovine serum albumin as the standard.

### Plant material

Three rice cultivars were grown. Koshihikari (Kurotani et al., 2015) and Azucena (Platten et al., 2013) are salt-sensitive. According to Ho et al. (2016), IR64 is moderately salt-tolerant cultivar, having a score of 5.75 in the Standard Evaluation System (SES) of the International Rice Research Institute (IRRI), where a score of 1.0 is tolerant, and a score of 9.0 is highly susceptible. On the other hand, Xie et al. (2020), classify IR64 as salt-tolerant.

Rice seeds were superficially sterilized by washing with 1% sodium hypochlorite for 5 minutes, followed by 3 washes with sterile water for 30 seconds and a final wash for 5 minutes, and then incubated at 30°C for 96 hours in the dark, under humid conditions in Petri dishes. Germinated seed were transplanted into Yoshida´s nutrient solution (Yoshida et al., 1976) and grown in hydroponic culture in a phytotron with a photoperiod of 16 hours day/8 hours night and a relative humidity of 70-80%. The nutrient solution was changed every 2 days and the pH was adjusted with 2 M NaOH to pH 5.6 - 5.8. To induce salt stress, NaCl solution was added to the medium to a final concentration of 200 mM (Liu et al., 2009), in the seedling stage, 17 days after germination. Leaves were harvested at 0, 6, 12 and 24 hours of treatment, placed into labeled plastic bags in a Dewar flask containing liquid nitrogen, and then stored in a liquid nitrogen tank. For each time point, three replica plant samples were collected for Koshihikari and Azucena, and two replica plant samples were collected from IR64.

### Metabolomics

Samples for metabolics measurements were prepared according to the protocol provided by the VIB Metabolomics Expertise Center (Leuven, Belgium) as follows. Porcelain mortars and pestles were pre-cooled at -80°C. Liquid nitrogen and the leaf sample were added to the mortar, and after grinding for 1 min, the tissue powder was transferred with a cold spatula to a tared, sterile 1.5 mL microtube in a cold labtop cooler, and the tube was weighed in a microbalance to obtain an approximation of the mg of tissue. The tissue powder was extracted by adding 80% methanol, previously cooled overnight at -80°C, at a volume of 500 µL per ≈ 50 mg tissue, and vortexing for 1 min. Methanol extracts were stored at -80°C overnight, then centrifuged 20,000 x *g* for 15 min at 4°C, and supernatants were transferred to microtubes, and dried using a SpeedVac. Dried samples were sent to the VIB Metabolomics Expertise Center in a styrofoam shipping container with dry ice and blue ice by express courier service, and samples were found to be cold upon arrival.

To measure the protein concentrations in the pellets that remained after methanol extraction, 200 µL of 200 mM NaOH were added to each pellet, the tubes were heated for 40 minutes at 80°C, cooled on ice, and centrifuged at 2,000 x *g* for 10 min. Protein concentrations of 10 µL of the supernatants were measured using the bicinchoninic acid protein assay (Pierce).

Mass Spectrometry measurements were performed were performed VIB Metabolomics Expertise Center using a Dionex UltiMate 3000 LC System (Thermo Scientific Bremen, Germany) coupled *via* heated electrospray ionization to a Q Exactive Orbitrap to a Q Exactive Orbitrap mass spectrometer (Thermo Scientific). 10 μl sample was taken from an MS vial and injected onto a C-18 column (Acquity UPLC -HSS T3 1. 8 μm; 2.1 x 150 mm, Waters). A step gradient was carried out using solvent A (10 mM TBA and 15 mM acetic acid in MilliQ water) and solvent B (100% methanol). The gradient started with 5% of solvent B and 95% solvent A and remained at 5% B until 2 min post injection. A linear gradient to 37% B was carried out until 7 min and increased to 41% until 14 min. Between 14 and 26 minutes the gradient increased to 95% of B and remained at 95% B for 4 minutes. At 30 min the gradient returned to 5% B. The chromatography was stopped at 40 min. The flow was kept constant at 0.25 mL/min and the column was placed at 40°C throughout the analysis. The MS operated in full scan mode (ranges [70.0000-1050.0000] and [300 – 850]) and negative mode using a spray voltage of 4.8 kV, capillary temperature of 300°C, sheath gas at 40, auxiliary gas at 10, the latter heated to 260°C. Automatic Gain Control (AGC) target was set at 3.0E+006 using a resolution of 140000. Data collection and analysis was performed using the Xcalibur software (Thermo Scientific).

## Results

### The short DHPD sequence identified in plants lacks cofactor and reductant binding sites found in mammalian DHPDs

**Figure 1:**
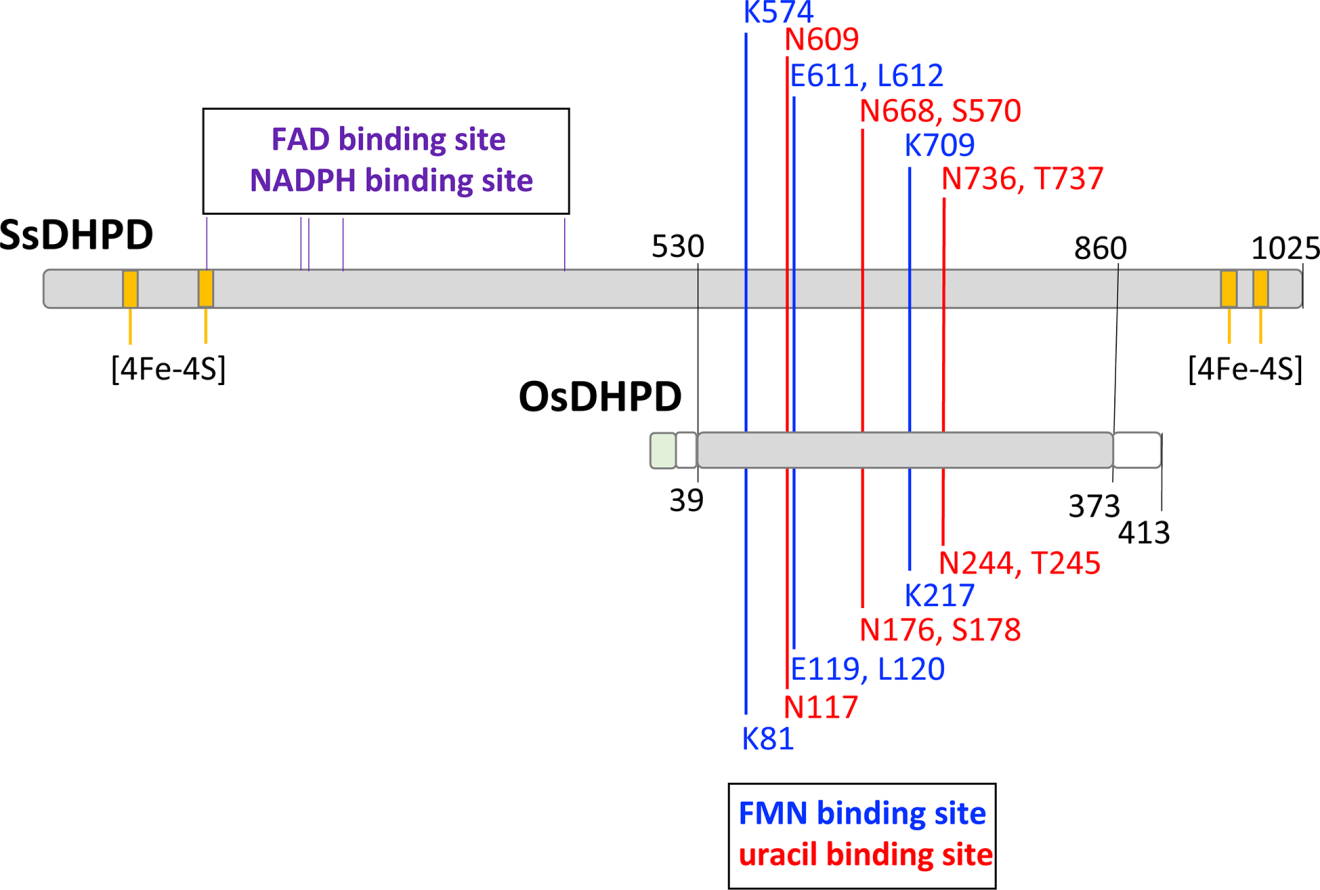
A schematic alignment of the mammalian DHPD and OsDHPD. The upper bar represents SsDHPD (Genbank U09179.2), and shows approximate locations of residues involved in binding cofactors and substrate, that are identified in the crystal structure (Dobritzsch et al., 2001). Position of residues interacting with FMN are indicated with vertical blue lines, positions of residues interacting with substrate are shown with vertical red lines, and the positions of four iron sulfur cluster are shown in yellow. The positions of residues involved in FAD and NADPH binding are indicated by the vertical black lines above the bar. The lower bar represents OsDHPD, and the region of homology with SsDHPD is highlighted in gray. The conserved FMN and uracil binding residues are shown below the bar. Regions that are not homologous are shown in white and green, the latter indicating the chloroplast targeting sequence.

An expression vector containing a full-length OsDHPD coding sequence, modified with an N-terminal histidine tag, was transformed into *E. coli*. Upon induction of expression, the recombinant protein in the cell lysate was found to be insoluble. TargetP 2.0 predicted a chloroplast transfer peptide with a cleavage site between residues R30 and A31, with a likelihood of 0.95. To increase solubility of the recombinant protein, we constructed a truncated version, OsDHPD-T, lacking 39 residues on the N-terminus. The site of the truncation was based on an alignment of the OsDHPD sequence with DHPDs from mammals and plants (**Supplementary Figure 1**), and included the first conserved region common to all the sequences. The histidine-tagged recombinant protein was purified using a Co^2+^ affinity column. Although OsDHPD-T was soluble, we were unable to demonstrate activity for the recombinant OsDHPD-T, with uracil and NADPH or NADH in the biological direction, or with dihydrouracil and NADP^+^ or NAD^+^ in the reverse direction. OsDHPD-T was also inactive in an assay for class 1A DHODHs, using dihydroorotate as the substrate and fumarate as the electron acceptor in the presence of 2,6‐dichlorophenol‐indophenol (DCIP). There are several possible explanations for the lack of activity of the truncated recombinant protein in the *in vitro* assays. The N-terminal sequence of the native protein in the chloroplast is not known, and the truncation of the protein may have caused inactivity. Another possibility is that the tag on the N-terminus might have interfered with the recombinant protein’s activity. A third, likely, possibility is that an additional protein or proteins of the chloroplast are needed to catalyze the first step of pyrimidine catabolism in plants (Zrenner et al., 2009; Cornelius et al., 2011). This would be consistent with the observation that the DHPDs of plants are approximately half the size of the mammalian enzymes, and although they conserve the FMN and uracil binding sites, they lack a binding site for the electron donor, NADPH, that is required for the reduction reaction in mammalian DHPD. The binding sites for FAD and 4Fe-4S clusters found in the C-terminal half of mammalian DHPD are also missing (**Figure 1**, **Supplementary Figure 1**).

### The plant catabolic DHPD is distinct from the biosynthetic dihydroorotate dehydrogenase

The DHPD from plants, a catabolic enzyme, has been misidentified as a biosynthetic DHODH (Liu et al., 2009), which catalyzes a similar redox reaction, but in the opposite direction. This error has persisted in the literature (Rolly et al., 2019; Ganie et al., 2019; Rolly et al., 2020). Higher eukaryotes, including plants, have class 2, mitochondrially-associated, biosynthetic DHODHs (EC 1.3.5.2) (Doremus and Jagendorf, 1985; Witz et al., 2012; Witte and Herde, 2020). Pairwise amino acid sequence alignments show that OsDHPD exhibits sequence identities of 41% with *E. coli* DHPD PreA, 38% with mammalian DHPD, 23 – 27% with class 2 DHODHs, and 26 – 28% with class 1A (EC 1.3.98.1), and class 1B (EC 1.3.1.14) DHODHs (**Table 1**).

**Table 1:** Percent identities in pairwise amino acid sequence alignments of DHPDs and DHODHs from different organisms.

	AtDHPD	SsDHPD	EcPreA	OsDHODH	AtDHODH	HsDHODH	TcDHODH	LlDHODH	LlDHODH
OsDHPD	83.3 (384)	38.4 (341)	41.2 (325)	23.3 (360)	24.9 (205)	24.5 (330)	26.9 (323)	27.6 (326)	23.2 (69)
AtDHPD		37.2 (360)	41.5 (360)	25.5 (208)	23.7 (371)	24.5 (383)	25.9 (324)	28.4 (324)	25.4 (71)
SsDHPD			31 (477)	22.9 (349)	23.1 (334)	23.4 (312)	30.9 (191)	30.3 (317)	22 (205)
EcPreA				24.3 (222)	25.3 (221)	24.2 (327)	27.8 (227)	28.3 (322)	25.3 (87)
OsDHODH					80.1 (428)	53.3 (379)	24.6 (342)	26.1 (337)	22.6 (53)
AtDHODH						52.8 (334)	24.9 (342)	24.4 (340)	22.9 (48)
HsDHODH							27 (319)	28.9 (315)	27.3 (55)
TcDHODH								27.4 (318)	29.1 (55)
LlDHODH PyrDB									24.5 (110)

DHPDs: OsDHPD, O.sativum, DQ102485, 414 residues; AtDHPD, Arabidopsis thaliana, AT3G17810, 426 residues; SsDHPD, Sus scrofa, U09179.2, 1026 residues; EcPreA, Escherichia coli, AAC75208.2, 411 residues; DHODHs: OsDHODH, class 2, O.sativum, DQ269457.1, 469 residues; AtDHODH, class 2, A. thaliana, At5g23300, 460 residues; HsDHODH, class 2, Homo sapiens, BC065245.1, 395 residues, TcDHODH, class 1A, Trypanosoma cruzi, EAN87213.1, 314 residues, LlDHODH, class 1B, PyrDb, Lactobacillus lactis, CAL97700.1;, 311 residues; LlDHODH, class 1B, PyrK, Lactobacillus lactis, PDB: 1EP3_B, 262 residues.

Pairwise alignments were performed with Lalign (Huang and Miller, 1991; https://www.ebi.ac.uk/Tools/psa/lalign/). Percent identities are shown, with the number of overlapping residues in parenthesis (in some cases the overlap includes gaps in the shorter of two sequences).

**Figure 2:**
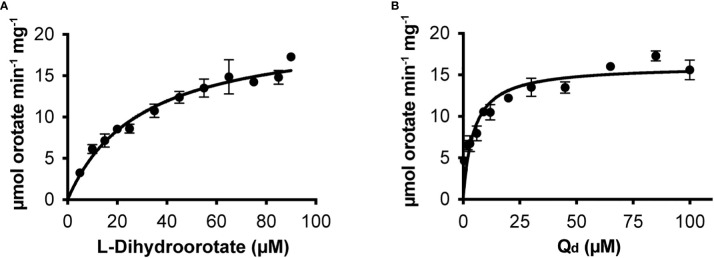
Steady state kinetics of purified recombinant OsDHODH. **(A)** Saturation curve for L-dihydroorotate. **(B)** Saturation curve for decylubiqinone, Q_d_.

We cloned the Class 2 OsDHODH from rice, and used the *E. coli* expression system to produce a truncated recombinant protein lacking 82 residues on the N-terminus that contain mitochondrial targeting and membrane associated sequences. It is important to note that Class 2 DHODHs from eukaryotic organisms are usually produced as truncated recombinant proteins (Hortua Triana et al., 2012), and the lack of these N-terminal sequences appear to have little effect on the kinetic parameters of the recombinant enzymes (Ullrich et al., 2002). We purified OsDHODH, and compared its kinetic parameters to those of other class 2 plant biosynthetic DHODHs (Ullrich et al., 2002; Garavito et al., 2019) (**Figure 2**, **Table 2**). The apparent K_m_s for dihydroorotate and the specific activities of the OsDHODH and *Solanum tuberosum* DHODH (Garavito et al., 2019) are quite similar, however the apparent K_m_ for decylubiquinone (Q_d_) is 6-fold lower in the rice enzyme. Both plant enzymes exhibit 5-fold lower specific activities compared to the human enzyme. These data support the idea that the catabolic DHPD and the biosynthetic DHODH play distinct roles in plants.

**Table 2:** Apparent kinetic parameters of dihydroorotate dehydrogenases from different organisms.

K_m_^app^ dihydroorotate (µM)	K_m_^app^ Q_d_ (µM)	apparent specific activity (µmol min^-1^ mg^-1^)	Organism
10	14	110	*Homo sapiens* ^1^
20	30	20	*Solanum* *tuberosum* ^2^
121	341	107	*Arabidopsis thaliana* ^3^
29.7 ± 3.5	4.56 ± 0.67	20.8 ± 1.0	*Oryza sativa* ^4^

^1^ Ullrich et al. (2001).^2^ Garavito et al. (2019). ^3^ Ullrich et al. (2002). ^4^This work. All activities were measured at 30°C.

### Recombinant β-UP protein exhibits a catalytic efficiency similar to the efficiencies of enzymes of secondary metabolism

**Figure 3:**
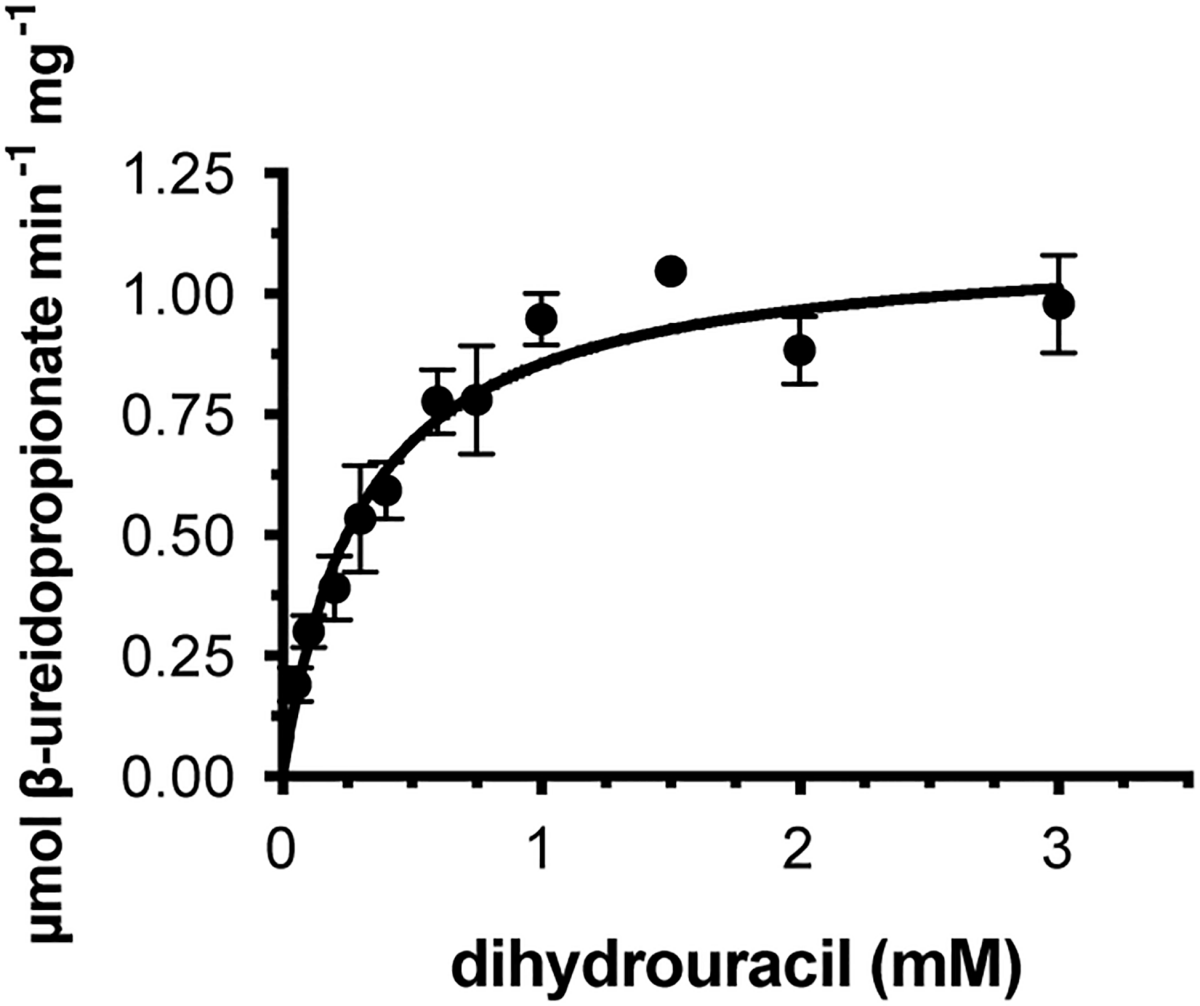
Steady state kinetics of purified recombinant OsDHP-T1. Saturation curve for dihydrouracil. Data were fitted to the Michaelis-Menten equation, v = (V_max_ · [S])/(K_m_ + [S]).

We cloned the coding sequences corresponding to the second and third enzymes in the rice pyrimidine catabolic pathway, and used the previously mentioned expression and purification methods to produce recombinant proteins. OsDHP is a 539-residue protein with a calculated molecular mass of 57,989 Da. It shares a 487-residue overlap, exhibiting 49% identity with the enzyme from *Bos taurus* (accession NP_001179143.3). OsDHP has a targeting sequence at the N-terminus that is similar to that found in the enzyme from *A. thaliana*, which has been localized the secretory system (Zrenner et al., 2009) (**Supplementary Figure 2**). The full-length OsDHP recombinant protein was insoluble. Two N-terminal truncations were produced to remove targeting sequence, OsDHP-T1 starting with residue 30 (EFCA), and OsDHP-T2 starting with residue 44 (GGDG). Both truncated recombinant proteins were soluble, but OsDHP-T2 was inactive, despite including all sequences conserved between plants and mammals (**Supplementary Figure 2**). OsDHP-T1 appeared to follow Michaelis-Menten kinetics, exhibiting the following apparent kinetic parameters, K_m_^app^ = 0.30 ± .04 mM for 5, 6-dihydrouracil, apparent specific activity = 1.10 ± 0.04 µmol·m^-1^·mg^-1^, k_cat_^app^ 1.07± 0.04 s^-1^, and a specificity constant, k_cat_^app^/K_m_^app^ = 3.6 x 10^3^ (**Figure 3**, **Table 3**). The efficiency of this recombinant enzyme, as measured by its apparent specificity constant, is two orders of magnitude lower than those of the mammalian enzymes. The N-terminal sequences of the active protein *in vivo* is unknown, and it is possible that the truncation may prevent optimal activity.

**Table 3:** Apparent kinetic parameters of dihydropirimidinases from different organisms with the substrate 5,6-dihydrouracil.

K_m_^app^ (mM)	apparent specific activity (µmol min^-1^ mg^-1^)	k_cat_^app^ (s^-1^)	k_cat_^app^/K_m_^app^ (s^-^1 M^-1^)	Organism
0.008	10.3	9.7	1.2 x 10^6^	*Bos taurus* ^1^*
0.019	14.8	13.3	7.0 x10^5^	Rattus norvegicus^2^**
0.30 ± 0.04	1.10 ± 0.05	1.07± 0.04	3.55 x 10^3^	Oryza sativa^3^** ^(OsDHP-T1)^

^1^ Brooks et al. (1983). ^2^ Kikugawa et al. (1994). ^3^This work. *Activity was measured at 30°C. **Activity was measured at 37°C.

**Figure 4:**
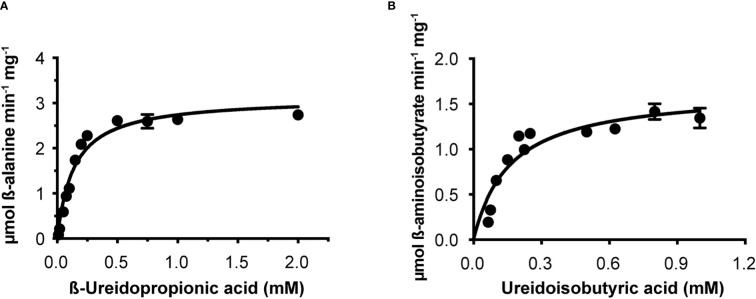
Steady state kinetics of purified recombinant Osß-UP. **(A)**, Saturation curve for ß-ureidopropionate. **(B)**, Saturation curve for ß-ureidoisobutyric acid. Data were fitted to the Michaelis-Menten equation, v = (V_max_ · [S])/(K_m_ + [S]).

Osß-UP is a 413-residue protein with a calculated molecular mass of 45,713 Da. It shares a 387-residue overlap, exhibiting 57% identity with the enzyme from *Bos taurus* (accession NP_001094520.1). An alignment with the enzyme from *A. thaliana* (accession NP_201242.2) exhibits a 402-residue overlap, with 81% identity. Osß-UP has been localized to the cytosol (Zrenner et al., 2009), and the full-length recombinant protein was soluble. Osß-UP appeared to follow Michaelis-Menten kinetics, exhibiting the following apparent kinetic parameters, K_m_^app^ = 0.14 ± .01 mM for ß-ureidopropionate, apparent specific activity = 3.12 ± 0.08 µmol·m^-1^·mg^-1^, k_cat_^app^ 2.54 s^-1^ ± 0.07 s^-1^, and a k_cat_^app^/K_m_^app^ = 1.8 x 10^4^ (**Figure 4**, **Table 4**). Apparent kinetic parameters for Osß-UP with ureidoisobutyric acid, resulting from the degradation of thymine, were, K_m_^app^ = 0.16 ± .02 mM, apparent specific activity = 1.67 ± 0.09 µmol·m^-1^·mg^-1^, k_cat_^app^ 1.36 ± 0.07 s^-1^, and a k_cat_^app^/K_m_^app^ = 8.5 x 10^3^ (**Figure 4**, **Table 5**). The specificity constants of Osß-UP with either of its two substrates are similar to those observed for the few ß-UP enzymes from other organisms that have been kinetically characterized, and they are also similar to the median values found in a survey of enzymes of secondary metabolism (2.5 s^-1^ for k_cat_, and 6.3 x10^4^ s^-1^M^-1^ for k_cat_/K_m_) (Bar-Even et al., 2011).

**Table 4:** Apparent kinetic parameters of ß-ureidopropionases from different organisms with the substrate ß-ureidopropionic acid.

K_m_^app^ (mM)	apparent specific activity (µmol min^-1 ^mg^-1^)	k_cat_^app^ (s^-1^)	k_cat_^app^/K_m_^app^ (s^-^1 M^-1^)	Organism
0.008	0.88	0.65	8.1 x 10^4^	*Rattus norvegicus* ^1^
0.019	–	0.31	1.6 x 10^4^	*Homo sapiens* ^2^
0.011	–	–	–	*Zea mays* ^3^*
0.006	0.47	0.37	6.1 x 10**^4^**	*Arabidopsis thaliana* ^4^**
2.1	–	26	1.2 x 10**^4^**	*Agrobacterium tumefaciens* ^5^*
0.14 ± 0.01	3.12 ± 0.08	2.54 ± 0.07	1.81 x 10^4^	*Oryza sativa* ^6^**

^1^ Traut (2000), ^2^ Maurer et al. (2018). ^3^ Walsh et al. (2001), ^4^ Andersen et al. (2008), ^5^ Martínez-Gómez et al. (2009), ^6^This work. *Activity was measured at 30°C. **Activity was measured at 25°C. The mammalian enzyme activities were measured at 37°C. The symbol “-“ indicates that data are not available.

**Table 5:** Apparent kinetic parameters of ß-ureidopropionases from different organisms with the substrate ureidoisobutyric acid.

K_m_^app^ (mM)	apparent specific activity (µmol min^-1 ^mg^-1^)	k_cat_^app^ (s^-1^)	k_cat_^app^/K_m_^app^ (s^-^1 M^-1^)	Organism
0.006	–	–	–	*Rattus norvegicus* ^1^
0.006	–	–	–	*Zea mays* ^2**^
6.6	–	24.	3.7 x 10**^3^**	*Agrobacterium tumefaciens* ^3*^
0.16 ± 0.02	1.67 ± 0.09	1.36 ± 0.07	8.5 x 10^3^	*Oryza sativa* ^4**^

^1^ Traut (2000), ^2^ Walsh et al. (2001), ^3^ Martínez-Gómez et al. (2009), ^4^This work. *Activity was measured at 30°C. **Activity was measured at 25°C. The mammalian enzyme activity was measured at 37°C. The symbol “-“ indicates that data are not available.

### Levels of *O. sativa* pyrimidine catabolic pathway intermediates show insignificant changes upon exposure to short-term salt stress

**Figure 5:**
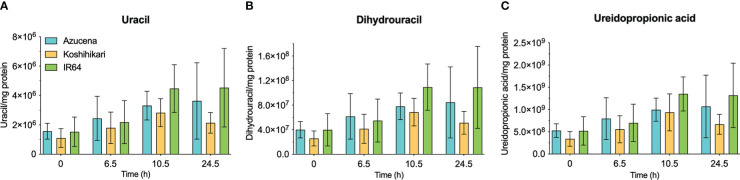
Levels of selected metabolites of pyrimidine catabolism in rice leaves during 24 hours of exposure to salt stress in salt-sensitive and salt-tolerant cultivars. Three rice cultivars were grown hydroponically in a phytotron, and 200 mM NaCl was added to growth media on day 17. Koshihikari and Azucena are salt-sensitive, and IR64 is salt-tolerant. Leaves were harvested during a 24-hour period post-treatment, and the levels of selected metabolites of pyrimidine catabolism, uracil **(A)**, dihydrouracil **(B)**, and ureidopropionic acid **(C)**, were measured. Values on the y-axis represent the integrated area of peaks associated with the metabolite of interest, normalized to mg protein in the sample. It is important to note that the levels of different compounds cannot be compared. No significant differences in metabolite levels were observed in a two-way ANOVA using GraphPad Prism 9.

**Figure 6:**
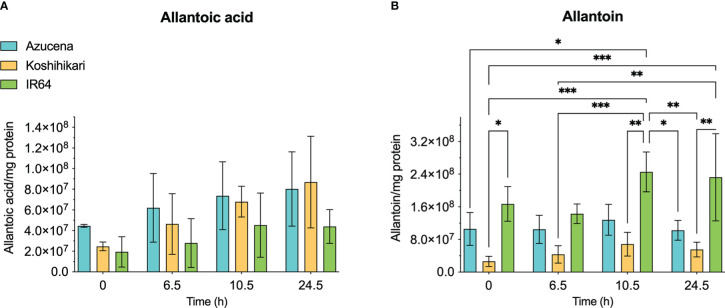
Levels of selected metabolites of purine catabolism in rice leaves during 24 hours of exposure to salt stress. Three rice cultivars were grown hydroponically in a phytotron, and 200 mM NaCl was added to growth media on day 17. Koshihikari and Azucena are salt-sensitive, and IR64 is salt-tolerant. Leaves were harvested during a 24-hour period post-treatment, and the levels of metabolites were measured. Allantoin **(B)** and allantoic acid **(A)** are the products of the fourth and fifth steps, respectively, of xanthine degradation. Values on the y-axis represent the integrated area of peaks associated with the metabolite of interest, normalized to mg protein in the sample. It is important to note that the levels of different compounds cannot be compared. Data were analyzed in a two-way ANOVA using GraphPad Prism 9 (*P values 0.01 - 0.05, ** P values 0.001 – 0.01, *** P values 0.0001 – 0.001).

The reports of the stress-induced increase in transcript levels of OsDHPD (Liu et al., 2009) led us to ask whether there was a concomitant increase of pathway intermediates. To address this question, we grew Koshihikari (Kurotani et al., 2015) and Azucena, (Platten et al., 2013), two salt-sensitive rice cultivars, as well as IR64, a salt-tolerant cultivar, hydroponically in a phytotron, and added 200 mM NaCl to the growth media at 17 days. Leaves were harvested during a 24-hour period post-treatment, and the levels of selected metabolites of pyrimidine catabolism (uracil, dihydrouracil, and ureidopropionic acid), were measured (**Figure 5**). No statistically significant differences in these metabolite levels were observed. We also measured two metabolites of purine catabolism, allantoin and allantoic acid (**Figure 6**), because accumulation of these ureide compounds is proposed to protect plants against oxidative stress by scavenging reactive oxygen species (Werner and Witte, 2011; Soltabayeva et al., 2022), or by activating ABA metabolism (Watanabe et al., 2014). Since dihydrouracil and ureidopropionic acid are also ureides, it is possible that they might play similar roles in ameliorating the oxidative stress caused by high salinity. No statistically significant differences were observed in the levels of allantoic acid in the samples, however, allantoin levels were significantly higher in samples from IR64, the salt-tolerant cultivar, compared to the samples from the salt-sensitive Koshihikari (**Figure 6**).

## Discussion

In plants, the principal functions of the reductive pyrimidine catabolic pathway are thought to be the maintenance of pyrimidine homeostasis, and the recycling of nitrogen under conditions of nitrogen limitation, as was concluded from studies on Arabidopsis (Zrenner et al., 2009; Cornelius et al., 2011). In rice, an additional role appears to be played by the pathway under conditions of abiotic stress, such as high salinity (Liu et al., 2009).

The first enzyme of the pyrimidine catabolic pathway, DHPD, is well-studied in mammals, and the crystallographic structure of the 1025-residue pig enzyme (SsDHPD) reveals binding sites for FAD and for the electron donor NADPH in the N-terminal half of the protein, and binding sites for FMN and uracil in the C-terminal half of the protein (Dobritzsch et al., 2001 **) (** **Figure 1**, **Supplementary Figure 1**). Additionally, there are binding sites for two iron sulfur clusters at the N-terminus, and another two iron sulfur clusters at the C-terminus (Dobritzsch et al., 2001 **) (** **Figure 1** **).** The DHPDs of plants are approximately 400 residues in size, and exhibit homology to the C-terminal half of the mammalian enzyme. Residues binding FMN and uracil are conserved (**Figure 1**). The last 40 residues on the C-terminus of OsDHPD align poorly with the SsDHPD sequence, and the latter extends 300 residues further, and includes sequences binding two iron sulfur clusters. It is not clear how the plant DHPDs achieve reduction of the uracil substrate in the absence of a binding site for a reductant, such as NADPH. The existence of an additional chloroplast protein that participates in the catalysis of the first step in the pathway has been postulated (Zrenner et al., 2009; Cornelius et al., 2011).

There is precedent for DHPD enzymes composed of two polypeptides in bacteria. The *E. coli* enzyme (EcDHPD) (EC 1.3.1.1) is a heterotetramer containing two PreT and two PreA subunits, homologous to the N- and C-terminal halves of mammalian DHPD, respectively (Hidese et al., 2011). Each subunit of the EcDHPD contains iron sulfur clusters, however the cosubstrate is NADH instead of NADPH (Hidese et al., 2011). Using Protein BLAST, we searched the plant databases for sequences of independent polypeptides with similarity to the N-terminal half of SsDHPD or to *E. coli* PreT, but without success.

There is confusion in the literature (Liu et al., 2009; Rolly et al., 2019; Rolly et al., 2020) between the catabolic plant DHPD enzyme, and the enzyme of the *de novo* biosynthetic pathway, DHODH, which catalyzes a similar redox reaction, but in the opposite direction. The observation that OsDHPD shares higher percent identities in pairwise sequence alignments with other DHPDs than it does with DHODHs of classes 2, 1A, or 1B (**Table 1**), the conservation of the SsDHODH FMN and uracil binding residues in OsDHPD, together with our activity measurements of recombinant OsDHODH (**Figure 2**, **Table 2**), clearly show that the catabolic DHPD and the biosynthetic DHODH are different enzymes.

This is the first report of kinetic parameters for the second and third enzymes of the pathway in plants. OsDHP-T1 exhibited K_m_^app^ ≈ 40-fold higher and a k_cat_^app^ ≈ 9-fold lower than the parameters of the enzyme from *B. taurus*. Indeed, the k_cat_^app^ and k_cat_^app^/K_m_^app^ are 2-fold, and 18-fold lower, respectively, than the median values found in a survey of enzymes of secondary metabolism (2.5 s^-1^ for k_cat_, and 6.3 x10^4^ s^-1^M^-1^ for k_cat_/K_m_) by Bar-Even et al. (2011) (**Table 3**). If the kinetic parameters of the truncated recombinant protein indeed reflect the *in vivo* properties of the native enzyme, the OsDHP would appear to be a low-efficiency enzyme, nevertheless, other truncation constructs should be tested. The recombinant Osß-UP protein had values of k_cat_^app^ and k_cat_^app^/K_m_^app^ close to the median values found for enzymes of secondary metabolism (Bar-Even et al., 2011) for the substrates ß-ureidopropionic acid and ureidoisobutyric acid (**Tables 4**, **5**).

Various studies have suggested that the plant pyrimidine degradative pathway may participate in the response to salinity and drought. It is important to keep in mind that abiotic stress resistance is a complex trait controlled by many genetic loci (Quan et al., 2018), thus the contribution of the pathway to abiotic stress tolerance would be one of many responses (van Zelm et al., 2020). Liu and co-workers observed an increase in DHPD transcripts within 24 h after subjecting rice to 200 mM NaCl, to drought, or to high temperature (Liu et al., 2009). On a longer time scale (12 d), overexpression of the enzyme caused transgenic plants to become more salt tolerant, while plants with DHPD knockdowns were more sensitive to salt (Liu et al., 2009). Since this initial observation, various studies have shown increased DHPD transcription in rice under abiotic stress (Shankar et al., 2016; Zhou et al., 2016; Li et al., 2018). DHPD is one of seven genes that were simultaneously selected for salt tolerance during domestication of rice, that were identified by Lv et al. (2022) in transcriptomics and genome wide association studies of wild and domesticated Asian and African rice. Narsai and coworkers found that in rice, DHPD was one of 10% - 14% of differentially expressed genes upregulated in response to salt and drought that was also upregulated in *A. thaliana* (Narsai et al., 2010). This gene was identified as a conserved upregulated drought-adaptive gene in a meta-analysis of *A. thaliana*, rice, wheat, and barley (Shaar-Moshe et al., 2015). Interestingly, no salt-induced increased expression levels for the second and third enzymes of the pathway have been reported.

Additional support for the participation of DHPD in plants’ response to salinity, is that expression in both *O. sativa* (Liu et al., 2009) and in *A. thaliana* (Cornelius et al., 2011) are induced by ABA, the major phytohormone responsible for plants’ adaption to abiotic stress (Manna et al., 2021). Analysis of the promotor region of OsDHPD showed presence of not only an ABA-responsive element (ABRE), but also an inducer of CBF expression (ICE) and C-repeat/dehydration-responsive elements, the latter indicating probable control by dehydration responsive element binding protein transcription factors (TF) (Liu et al., 2009). More recently, Rolly and co-workers suggested that an ABRE in the promotor of AtDHPD is the putative site of bZIP TF binding (Rolly et al., 2020). In a further complexity, plant DHPDs may be involved in regulation of phytohormones. In *A. thaliana*, DHPD knockout plants exhibited a large increase in ABI1, a phosphatase regulator of ABA signaling, however the knockouts show only 15% of wild type expression of ABF4, a member of the bZIP TF family, which binds to ABA-responsive elements (Cornelius et al., 2011). Additionally, under drought conditions, a large increase in AtbZIP62 TF expression was observed in *A. thaliana* plants that have knockouts of AtDHPD (Rolly et al., 2020).

An upregulation of rice DHPD during abiotic stress might be reflected in accumulation of pathway intermediates or end products. There is little information on metabolite levels for short-term exposure to salt. Our results showed insignificant changes in metabolites derived from uracil after 24 h of salt stress. Liu and coworkers (2013) reported a modest increase of ~2-fold in 3-aminoisobutyric acid at 24 h, remaining stable through day 7, in salt-exposed rice cell suspension cultures. On the other hand, several metabolomic studies demonstrate accumulation of pathway metabolites in rice subjected to long-term salt exposure (≥ 7 days). The metabolite 5, 6-dihydrouracil is among the 372 differentially upregulated metabolites in a resistant line, where > 2-fold change was used as the threshold value (Wang et al., 2021). Wanichthanarak and co-workers (2020) identify ß-alanine as a discriminative metabolite (log2-fold change 2.15) in the leaves of rice exposed to salt stress for 9 days. Under conditions of severe drought, negligible changes in 3-aminoisobutyric acid occur initially, until days 26 and 36, when ≥ 10-fold increase are observed in both drought resistant and tolerant rice cultivars (Ma et al., 2016). Principal component analysis of the metabolomic profiles of roots showed that ß-alanine and 3-aminoisobutyric acid were the first and third components in the top 50 that were differentially overexpressed during polyethylene glycol-induced osmotic stress in resistant rice cultivars (Matsunami et al., 2021). It should be noted that ß-alanine is generated by the degradation of the polyamine spermidine and possibly during propionate metabolism (Parthasarathy et al., 2019), and 3-aminoisobutyric acid is a product of valine degradation (Kegg Pathway Database, https://www.genome.jp/kegg/pathway.html). ß-alanine is a substrate in the production of pantothenate, the osmolyte ß-alanine betaine, and homoglutathione (Parthasarathy et al., 2019).

As described above, evidence is accumulating that the pyrimidine catabolic pathway may play a role in the response to salinity. Transcriptomic studies suggest that upregulation of DHPD, the first enzyme in the pathway, confers an advantage under conditions of abiotic stress in rice, however there is no evidence that increased expression of the second or third enzymes occur under such conditions. As shown here, and by others (Liu et al., 2013), short-term exposure to salt appears to result in insignificant or modest changes in levels of pathway metabolites in rice. In contrast, several studies show that long-term exposure leads to elevated levels of some pathway metabolites. The apparent intervention of DHPD in the regulation of ABA (Cornelius et al., 2011; Rolly et al., 2020), raises the intriguing possibility that regulation by DHPD may confer an advantage for surviving abiotic stress, and this notion should be further investigated.

## Data availability statement

The original contributions presented in the study are included in the article/**Supplementary Material**. Further inquiries can be directed to the corresponding author.

## Author contributions

AL, HN-O, MR-B, and BZ conceived and designed the experiments. AL, HN-O, MR-B, DP and LS conducted the experiments. AL, HN-O, MR-B, DP and BZ analyzed the data. BZ wrote the manuscript. All authors reviewed the results and approved the final version of the manuscript.
