# Supplementary material for: New Insights into rice pyrimidine catabolic enzymes
Source: Front Plant Sci. 2023 Feb 1;14:1079778. doi: 10.3389/fpls.2023.1079778 (PMC9930899; doi:10.3389/fpls.2023.1079778)
Supplement: Supplementary file 1 [file DataSheet_1.pdf]

Supplementary Files

New Insights into rice pyrimidine catabolic enzymes.

Andrea J. Lopez[1,†,‡], Heidy Y. Narvaez-Ortiz[1,†,‡], Maria A. Rincon-Benavides[1,‡], Dania Camila Pulido[1,†], Luis Eduardo Fuentes Suarez[1], Barbara H. Zimmermann[1*].

[1]Departamento de Ciencias Biológicas, Universidad de los Andes, Carrera 1 # 18A – 10, Bogotá D.C., Colombia

† Shared first authorship.

‡ Present address: HYN-O, Institute of Molecular Biology, University of Oregon, 1229 University of Oregon, Eugene, OR, USA.; AJL, Department of Biomedicine, University of Bergen, Bergen, Norway; MAR-B, Department of Biomedical Engineering, The Ohio State University, Columbus, OH 43210, USA; DCP, Biozentrum, University of Basel, Basel, Switzerland.

***Correspondence:**
Barbara H. Zimmermann
bazimmer@uniandes.edu.co

1. **Supplementary Table 1. Primers used in this study.**
2. **Supplementary Figure 1. Alignment of dihydropyrimidine dehydrogenases (DHPDs)**
3. **Supplementary Figure 2. Alignment of dihydropyrimidinases (DHPs)**
4. **Supplementary Figure 3. Denaturing gel electrophoresis of recombinant protein of the reductive pyrimidine catabolic pathway of *O. sativa* purified from bacterial extracts**

Supplementary Table 1. Primers used in this study.

Enzyme from *O. sativum*	Primers †	Primer name	Description
Dihydropyrimidine dehydrogenase	5′-ggatcctATGGATTCGCTGACTCTCCG-3′	DHPD FORWARD	full length protein, forward
	5′-ggatcctATGGATTCGCTGACTCTCCG-3′	OsDHPD-T1for	truncated protein, forward
	5′-ggatccTCAGTTGGCCACCATGCTTT-3′	DHPDH REVERSE	reverse
Dihydropyrimidinase	5´-ctcgagATGGCGATGCCCTGCCG-3′	OsDHP-F	full-length protein, forward
	5′-ctcatcctcgagGAGTTCTGCGCGGCCGTC-3′	OsDHP-T1	truncation 1 protein, forward
	5′-ctcatcctcgagGACGGGAGGAGGATCCTGATCAG-3′	OsDHP-T2	truncation 2 protein, forward
	5′-TTGCTGTCCCTCAGCAACA-5′	OsDHP-RS	5′-end fragment, reverse
	5′-ATTCCAGTGAATGGCAATTTAACTG-3′	OsDHP-FS	3′-end fragment, forward
	5′-ctcgagTTAAGCAGCAGCCTTGCCACG-3´	OsDHP-R	reverse
ß-Ureidopropionase	5′-ctcatcctcgagGACGGGAGGAGGATCCTGATCAG-3′	OsBUP-For	full length protein, forward
	5′-ctcatcctcgagTTACGCACTCTTATTGATCAAGGGGT-3′	OsBUP-Rev	reverse
Dihydroorotate dehydrogenase	5′-ggatcctGATGAAGCCAAGTTTTGTGGC-3´	OsDHODH-F	truncated protein, forward
	5´-aggatccTCATTTGAAATCTGCCCCAA-3´	OsDHODH-R	reverse

†Lower case are additional sequences that include restriction sites.

CLUSTAL O(1.2.4) multiple sequence alignment

```
SsDHPD          -------------------------------KYIQAQYGASVSAKPELPLFYTPVDLVDI          529(533)
HsDHPD          -------------------------------KYVQSQYGASVSAKPELPLFYTPIDLVDI          529
EcPreA          ------------------------------------------------------MLTKDL          6
AtPYD1          MASMSFALNRFSGLSSKTTLSADFDPSSRRSFLPPTRVG--------LKISSAAESEPDL          52
OsDHPD          MESLT--------LRASPSTAAPLRRV-----PGRRHAAVS------V-RASAGAGEPDL          40
StDHPD          MASLGLAQRIRIGLDGNSGVEFPMMDRAGRLNFGKKRVGFR------VMASEGQSVEPDL          54
                                                                          *:

                                                        ↓
SsDHPD          SVEMAGLKFINPFGLASAAPTTSSSMIRRAFEAGWGFALTKTFSLDKDIVTNVSPRIVRG          589(593)
HsDHPD          SVEMAGLKFINPFGLASATPATSTSMIRRAFEAGWGFALTKTFSLDKDIVTNVSPRIIRG          589
EcPreA          SITFCGVKFPNPFCLSSSPVGNCYEMCAKAYDTGWGGVVFKTIGFFIA--NEVSPRFDHL          64
AtPYD1          SVTVNGLKMPNPFVIGSGPPGTNYTVMKRAFDEGWGAVIAKTVSLDASKVINVTPRYARL          112
OsDHPD          SVRVNGLKMPNPFVIGSGPPGTNYTVMKRAFDEGWGGVIAKTVSLDAEKVINVTPRYARL          100
StDHPD          SVTVNGLKMPNPFVIGSGPPGTNYTVMKRAFDEGWGGVIAKTVSLEADKVKNVTPRYAKL          114
                *: . *:*: *** :.*.   .   :  :*:: *** .: **..:      :*:**  :

                                ↓ ↓↓
SsDHPD          TTSG-PMYGPGQSSFLNIELISEKTAAYWCQSVTELKADFPDNIVIASIMCSYNKNDWME          648(652)
HsDHPD          TTSG-PMYGPGQSSFLNIELISEKTAAYWCQSVTELKADFPDNIVIASIMCSYNKNDWTE          648
EcPreA          VKE-----DTGFIGFKNMEQIAEHPLEENLAALRRLKEDYPDKVLIASIMGE-NEQQWEE          118
AtPYD1          RTGSNGSAKTDVIGWQNIELISDRPLETMLKEFERLKKEYPDRILIASVMEEYNKTAWEE          172
OsDHPD          RADPNGSTKSPIIGWQNIELISDRPLETMLNEFKQLKKEYPDRILIGSIMEEYNKAAWHE          160
StDHPD          RADANGSAKGQIIGWQNIELISDRPLETMLKEFKQLKEEYPDRILIASIMEEYNKAAWEE          174
                             .: *:* *:::        . .** ::**.::*.*:* . *:  * *

                               ↓ ↓                                    ↓
SsDHPD          LSRKAEASGADALELNLSCPHGMGERGMGLACGQDPELVRNICRWVRQAVQIPFFAKLTP          708(712)
HsDHPD          LAKKSEDSGADALELNLSCPHGMGERGMGLACGQDPELVRNICRWVRQAVQIPFFAKLTP          708
EcPreA          LARLVQEAGADMIECNFSCPQ-MTSHAMGSDVGQSPELVEKYCRAVKRGSTLPMLAKMTP          177
AtPYD1          LIDRVEQTGVDALEINFSCPHGMPERRMGAAVGQDCALLDEVCGWINAKATVPVWAKMTP          232
OsDHPD          LIERVEESGVDALEINFSCPHGMPERKMGAAVGQDCDLLEEVCGWINEKATVPVWAKMTP          220
StDHPD          LIYRCEETGIDAFEINFSCPHGMPERRMGAAVGQDCDLLEEVCGWINAVATVPVWAKMTP          234
                *    : :* * :* *:***: * .: **   **.  *: : *  :.    :*. **:**

                                       ↓↓
SsDHPD          NVTDIVSIARAAKEGGADGVTATNTVSGLMGLKADGTPWPAVGAGKRTTYGGVSGTAIRP          768(772)
HsDHPD          NVTDIVSIARAAKEGGANGVTATNTVSGLMGLKSDGTPWPAVGIAKRTTYGGVSGTAIRP          768
EcPreA          NIGDMCEVALAAKRGGADGIAAINTVKSITNIDLNQKIGM-PIVNGKSSISGYSGKAVKP          236
AtPYD1          NITDITEPARVSLKSGCEGIAAINTIMSVMGIDMKTLRPE-PCVEGYSTPGGYSYKAVRP          291
OsDHPD          NITDITKPARISLKSGCEGVSAINTIMSVMGINLKTLRPE-PCVEGYSTPGGYSARAVHP          279
StDHPD          NITDITKPARVALNQGCEGVSAINTIMSVMGINLDTLRPE-PCVEGYSTPGGYSSKAVHP          293
                *: *: . *  : . *.:*::* **: .: .:. .            :: .* *  *::*

SsDHPD          IALRAVTTIARAL------PGFPILATGGIDSAESGLQFLHSGASVLQVCSAVQNQDFTV          822(826)
HsDHPD          IALRAVTSIARAL------PGFPILATGGIDSAESGLQFLHSGASVLQVCSAIQNQDFTV          822
EcPreA          IALRFIQQ----MRTHPELRDFPISGIGGIETWEDAAEFLLLGAATLQVTTGIMQYGYRI          292
AtPYD1          IALAKVMNIAKMMKSEFSED-RSLSGIGGVETGYDAAEFILLGSNTVQVCTGVMMHGYGH          350
OsDHPD          IALAKVMQIARMMKEEFADG-QSLSAIGGVETGNDAAEFILLGADTVQVCTGVMMHGYGL          338
StDHPD          IALAKVMNIARMMKSEFGDKDYSLSAIGGVETGGDAAEFILLGADTVQVCTGVMMHGYGL          353
                ***  :       :           : . **:::  .. :*:  *: .:** :.:   .:

SsDHPD          IQDYCTGLKALLYLKSIEELQGWDGQSPGTESHQKGKPVPRIAELMGKKLPNFGPYLEQR          882(886)
HsDHPD          IEDYCTGLKALLYLKSIEELQDWDGQSPATVSHQKGKPVPRIAELMDKKLPSFGPYLEQR          882
EcPreA          VEDMASGLSHYLADQGFD------------------------------------------          310
AtPYD1          VKTLCAELKDFMKQHNFS------------------------------------------          368
OsDHPD          VKKLCAELQDFMRQHNFS------------------------------------------          356
StDHPD          VKTLCSELKDFMRKHNFS------------------------------------------          371
                ::  .: *.  :  :.:.

SsDHPD          KKIIAEEKMRLKEQNAAFPPLERKPFIPKKPIPAIKDVIGKALQYLGTFGELSNIEQVVA          942(946)
HsDHPD          KKIIAENKIRLKEQNVAFSPLKRNCFIPKRPIPTIKDVIGKALQYLGTFGELSNVEQVVA          942
EcPreA          ---------------------------------SLQEMVGLANNNIVPAEDLDRSYIVYP          337
AtPYD1          ---------------------------------TIEEFRGHSLQYFTTHTDLVKRQKE--          393
OsDHPD          ---------------------------------SIEDFRGASLPYFTTHTDLVHRQRE--          381
StDHPD          ---------------------------------SIEDFRGTSLEYFTTHTDLVRRQQE--          396
                                                 ::::. * :   :    :* .

SsDHPD          VIDEEMCINCGKCYMTCNDSGYQAIQFDPETHLPTV-TDTCTGCTLCLSVCPIIDCIRMV          1001(1005)
HsDHPD          MIDEEMCINCGKCYMTCNDSGYQAIQFDPETHLPTI-TDTCTGCTLCLSVCPIVDCIKMV          1001(1005)
EcPreA          RINLDKCVGCGRCYISCYDGGHQAMEWSEKTRTPHCNTEKCVGCLLCGHVCPVG-CIELG          396
AtPYD1          AVEQRKAEK---------RGLKSDKDWTGDGFVKET--ESMVSN----------------          426
OsDHPD          AINQRKAIR---------KGLESDKDWTGDGFVKET--ESMVSN----------------          414
StDHPD          AIRQRKAVK---------KGLQSDKDWTGDGFVQET--ESMVSN----------------          429
                 :     .              .  .  ::  .        :. ..

SsDHPD          SRTTPYEPKRGLPLAVNPVC   1021(1025)
HsDHPD          SRTTPYEPKRGVPLSVNPVC   1021
EcPreA          EVKFKK-GEKEHPVTL----   411
AtPYD1          --------------------   426
OsDHPD          --------------------   414
StDHPD          --------------------   429
```

Supplementary Figure 1. **Alignment of dihydropyrimidine dehydrogenases (DHPDs).** The alignment was performed with Clustal Omega (Madeira et al. 2022). The predicted cleavage site for the chloroplast transfer peptide for OsDHPD by Target P 2.0 is underlined. The arrows indicate the FMN and uracil binding sites (Also shown in Figure 1) identified in the crystal structure of the the *Sus scrofa* enzyme by Dobritsch et al. 2001. The bold letters show the start of the recombinant, truncated DHPD, OsDHPD-T.

SsDHPD, *Sus scrofa*, U09179.2, residues 501 to 1021. The numbering shown is according to the accession number, the numbering in parenthesis is according to Dobritsch et al., 2001 ; HsDHPD, *Homo sapiens*, isoform 1, NP_000101.2, residues 501 to 1021; EcPreA, *Escherichia coli*, AAC75208.2, complete sequence; AtDHPD, *Arabidopsis thaliana*, AT3G17810, complete sequence; OsDHPD, *Oryza sativum*, DQ102485, complete sequence; StDHPD, *Solanum tuberosum*, XP_006363983, complete sequence.

```
RnDHP      ------------------------------------------------MAPQERLLIRG     11
BtDHP      ------------------------------------------------MAARSLLLIRG     11
OsDHP      MAMPCRLHGDILLLLLGAVAVAVAHPAANEFCAAV-GGGSGGCGVGGGGGGDGRRILIRG     59
AtDHP      MALDAFFFIVSLFLLF---PSPSASESTTQFCSAGRENGVGSCGV------SSTRILIKG     51
                                                                  :**:*

RnDHP      GRVVNDDFSQVADVLVEDGVVRALGRDLLPPGDTSRGLRILDAAGKLVLPGGIDTHTHMQ     71
BtDHP      GCVVNDDSSQEADVLVEDGVVQAVGRHVLPPGDAPAGLRVLDAAGKLVLPGGIDTHTHMQ     71
OsDHP      GTVVNAHRVEEADVYVEDGVIVAVRPNI-PVGD--DHVKVIDATGKYVMPGGIDPHTHLE     116
AtDHP      GTVVNAHHQELADVYVENGIIVAVQPNI-KVGD--E-VTVLDATGKFVMPGGIDPHTHLA     107
           * *** .  : *** **:*:: *:  .:    **     : ::**:** *:***** ***:

RnDHP      FPFMGSQSVDDFHQGTKAALAGGTTMIIDFAIPQKGSSLIEAFETWRNWADPKVCCDYSL     131
BtDHP      FPFMGSRSVDDFHLGTKAALAGGTTMIMDFAIPHKGHSLIEAFDTWRSWADSQVCCDYSL     131
OsDHP      MEFMGTVTIDDFFSGHAAALAGGTTMHIDFVIPVNGN-LTAGLESYKQK-AEKSAMDYGF     174
AtDHP      MEFMGTETIDDFFSGQAAALAGGTTMHIDFVIPVNGN-LVAGFEAYENK-SRESCMDYGF     165
           : ***: ::***. *  ********* :**.** :*  *  .::::..     : . **.:

RnDHP      HVAVTWWSDKVKEEMKTLAQDKGVNSFKMFMAYKDLYMVQDQQMYAAFSQCKEIGAIAQV     191
BtDHP      HVAVTWWSDQVKEEMKILTQDKGVNSFKMFMAYKDVYMVRDVELYEAFSRCKEIGAIAQV     191
OsDHP      HMAITKWNDDVSREMETMVKEHGINSFKFFMAYKGSLMVTDDLLLQGLQKCKSLGALAMV     234
AtDHP      HMAITKWDEGVSRDMEMLVKEKGINSFKFFLAYKGSLMVTDDLLLEGLKRCKSLGALAMV     225
           *:*:* *.: *..:*: :.:::*:****:*:***.   ** *  :   .:.:**.:**:* *

RnDHP      HAENGDLIAEGAKKMLALGITGPEGHELCRPEAVEAEATLRAITIASAVNCPLYIVHVMS     251
BtDHP      HAENGDLVAEGAKKMLALGITGPEGHELSRPEAVEAEATLRAVTIASAVNCPLYVVHVMS     251
OsDHP      HAENGDAVAEGQQRMIDLGITGPEGHALSRPPVLEGEATARAIRLAKFVNTPLYVVHVMS     294
AtDHP      HAENGDAVFEGQKRMIELGITGPEGHALSRPPVLEGEATARAIRLARFINTPLYVVHVMS     285
           ****** : ** ::*: ********* *.** .:*.*** **: :*  :* ***:*****

RnDHP      KSAAKVIADAKREGKVVYGEPIAAGLGTDGTQYWNKEWRHAAHHVMGPPLRPDPSTPGFL     311
BtDHP      KSAAKVVADARRDGKVVYGEPIAAGLGTDGTHHWDREWLHAAHHVMGPPLRPDPSTPDFL     311
OsDHP      IDAMDEIAKAKREGQRVIGEPVVSGLVLDDSWLWDPDFMIASKYVMSPPIREAGHNKA-L     353
AtDHP      VDAMDEIAKARKSGQKVIGEPVVSGLILDDHWLWDPDFTIASKYVMSPPIRPVGHGKA-L     344
            .* . :*.*::.*: * ***:.:**  *.   *: ::  *:::**.**:*         *

RnDHP      MNLLANGDLTTTGSDNCTFNTCQKALGKDDFTKIPNGVNGVEDRMSVIWEKGVHSGKMDE     371
BtDHP      MNLLANDDLTTTGSDNCTFNSCQKALGKDDFTKIPNGVNGVEDRMSIIWEKGVHSGKMDE     371
OsDHP      QVALSSGILQLVGTDHCTFNSTQKAFGSDDFRKIPNGVNGIEERMHIIWDSMVETGRISV     413
AtDHP      QDALSTGILQLVGTDHCTFNSTQKALGLDDFRRIPNGVNGLEERMHLIWDTMVESGQLSA     404
              *:.. *  .*:*:****: ***:* *** :*******:*:** :**:. *.:*::.

RnDHP      NRFVAVTSTNAAKIFNLYPKKGRIAVGSDADMVIWDPEATRTISAKTHHQAVNFNIFEGM     431
BtDHP      NRFVAVTSTNAAKIFNLYPRKGRIAVGSDADIVIWDPKATRTISAKTHHQAVNFNIFEGM     431
OsDHP      SDYVRVTSTECAKIFNIYPRKGAILEGSDADIIILNPERSFVMGAHTHHSRSNTNVYEGR     473
AtDHP      TDYVRITSTECARIFNIYPRKGAILAGSDADIIILNPNSSYEISSKSHHSRSDTNVYEGR     464
           . :* :***:.*:***:**:** *  *****::* :*: :  :.:::**.  : *::**

RnDHP      VCHGVPLVTISRGRVVYEAGVFDVTAGHGKFIPRQPFAEFIYKRVKQRDQTC---TPIPV     488
BtDHP      VCHGVPVVTISRGKVVYEAGVFSVSAGDGKFIPRKPFAEYIYKRIKQQDQTC---IPTPV     488
OsDHP      KGKGMVEVTISRGRVVWEDGILNVVPGSGRYVRTPPYS-YLFDGIEKSDAAYRASLRAPV     532
AtDHP      RGKGKVEVTIAGGRIVWENEELKVVPRSGKYIEMPPFS-YLFDGIEKSDANYLSSLRAPV     523
             :*    ***: *::*:*   :.*    *:::   *:: :::. ::: *          **

RnDHP      KRAPYKGEVITLKPRETKEDDTAGTRMQGHS 519
BtDHP      KREPYKGEVITLTSKED---STHGTRKQAQP 516
OsDHP      KRGKAAA------------------------ 539
AtDHP      KRVRTEAT----------------------- 531
           **     .
```

Supplementary Figure 2. **Alignment of dihydropyrimidinasedehydrogenases (DHPs).** The alignment was performed with Clustal Omega (Madeira et al. 2022). The predicted cleavage site for a signal peptide predicted for OsDHP by SignalP-5.0 is underlined.
The bold letters show the start of the recombinant, truncated DHP, OsDHP-T1 (EFCA), and OsDHP-T2 (GGDG). Both recombinant proteins were soluble, but only OsDHP-T1 was active. RnDHP, *Rattus norvegicus*, D63704.1; BtDHP, *Bos taurus*, NM_001192214.3; OsDHP, *Oryza sativa* DHP, AK072454; AtDHP, *Arabidopsis thaliana* DHP, AF465755.1.

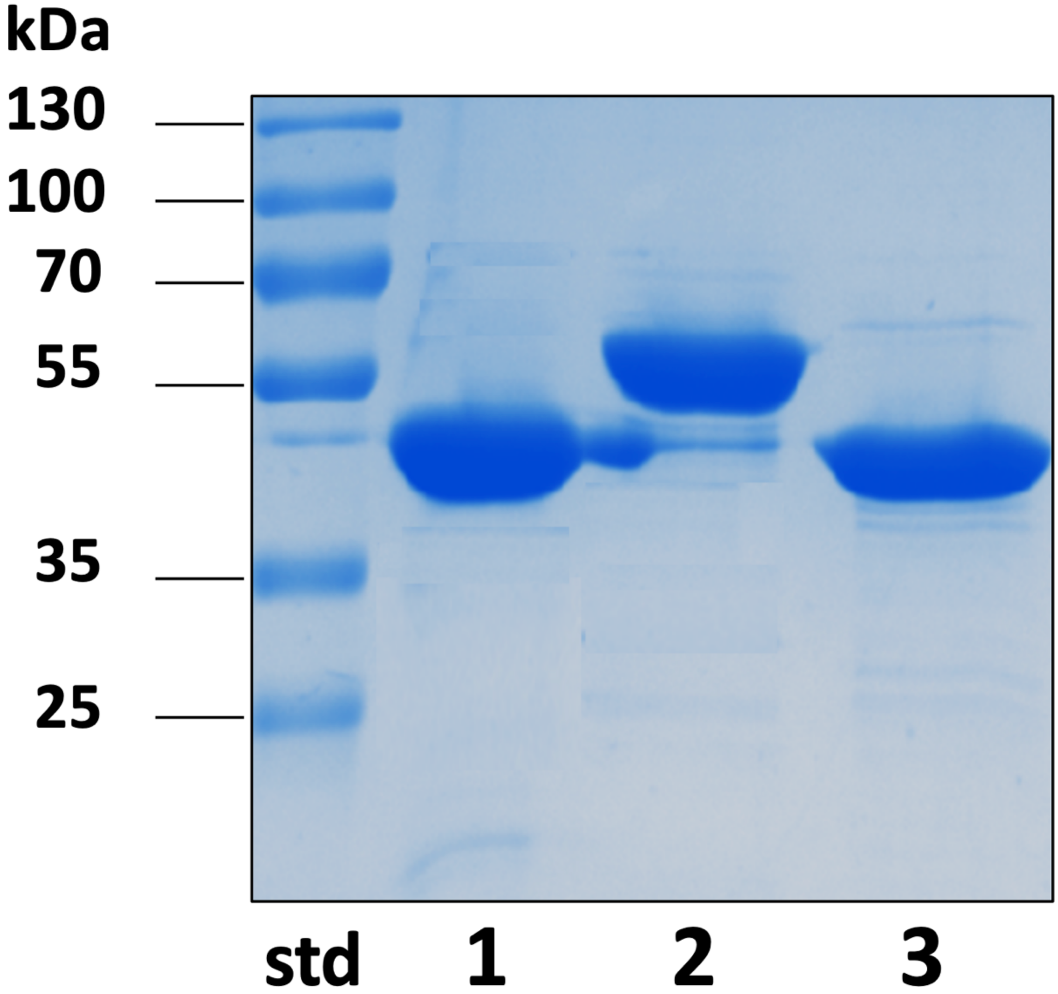

Supplementary Figure 3. Denaturing gel electrophoresis of recombinant protein of the reductive pyrimidine catabolic pathway of *O. sativa* purified from bacterial extracts. The pET19b expression system was used to produce recombinant, truncated OsDHPD-T (predicted molecular mass, 44,697 Da). An N-terminal his-tag precedes the OsDHPD sequence that begins with the residues LSVR, eliminating 39 residues of a chloroplast targeting sequence at the N-terminus. The pET19b expression system was also used to produce recombinant, truncated OsDHP-T1 (predicted molecular mass, 58,226 Da). An N-terminal his-tag precedes the OsDHP sequence that begins with the residues EFCA, eliminating 29 residues of a secretory system targeting sequence at the N-terminus. The pET15b expression system was used to produce recombinant, full-length Osβ-UP (predicted molecular mass, 48,267 Da). The recombinant proteins were purified using Co^{2+} resin.

REFERENCES

Dobritzsch D, Schneider G, Schnackerz KD, Lindqvist Y. Crystal structure of dihydropyrimidine dehydrogenase, a major determinant of the pharmacokinetics of the anti-cancer drug 5-fluorouracil. EMBO J. 2001 Feb 15;20(4):650-60. doi: 10.1093/emboj/20.4.650. PMID: 11179210

Madeira F, Pearce M, Tivey ARN, Basutkar P, Lee J, Edbali O, Madhusoodanan N, Kolesnikov A, Lopez R. Search and sequence analysis tools services from EMBL-EBI in 2022. Nucleic Acids Res. 2022 Apr 12;50(W1):W276-9. doi: 10.1093/nar/gkac240. PMID: 35412617
